# Host-Guest Chemistry in Layer-by-Layer Assemblies Containing Calix[*n*]arenes and Cucurbit[*n*]urils: A Review

**DOI:** 10.3390/polym10020130

**Published:** 2018-01-29

**Authors:** Uichi Akiba, Daichi Minaki, Jun-ichi Anzai

**Affiliations:** 1 Graduate School of Engineering and Science, Akita University, 1-1 Tegata Gakuen-machi, Akita 010-8502, Japan; uakiba@gipc.akita-u.ac.jp; 2 Graduate School of Pharmaceutical Sciences, Tohoku University, Aramaki, Aoba-ku, Sendai 980-8578, Japan; daichi.minaki.e3@tohoku.ac.jp

**Keywords:** layer-by-layer assemblies, host-guest complexation, calix[*n*]arene, cucurbit[*n*]uril

## Abstract

This review provides an overview of the synthesis of layer-by-layer (LbL) assemblies containing calix[*n*]arene (CA[*n*]) and cucurbit[*n*]uril (CB[*n*]) and their applications. LbL assemblies, such as thin films and microcapsules, containing selective binding sites have attracted considerable attention because of their potential use in separation and purification, sensors for ions and molecules, and controlled release. CA[*n*]-containing LbL films have been prepared using sulfonated CA[*n*] and cationic polymers to construct chemical sensors and molecular containers. CA[*n*]-containing LbL films deposited on the surface of a porous support are useful as ion-selective membranes that exhibit selective permeability to monovalent ions over multivalent ions. CB[*n*]s have been used as molecular glues for the construction of LbL films and microcapsules by taking advantage of the strong affinity of CB[*n*]s to aromatic compounds. CB[*n*]s form a stable 1:1:1 ternary complex with electron-rich and electron-deficient molecules in LbL films to stabilize the assemblies. CB[*n*]-containing LbL films can also be deposited on the surfaces of micro templates and nanopore membranes to construct microcapsules for controlled release and nanochannels for selective ion transport, respectively.

## 1. Introduction

Layer-by-layer (LbL) thin films are widely used for the development of film devices because they are simple to prepare and offer a wide choice of materials. LbL thin films were first prepared using synthetic polymers by alternate and repeated depositions on the surface of solid substrates through electrostatic interactions [1,2]. LbL deposition affords stratified multilayer architectures on the surface. A variety of interactions, such as hydrogen bonds [3,4], host-guest complexations [5], charge-transfer (CT) interactions [6], DNA hybridization [7], covalent bonds [8], and protein-ligand affinity [9] are available as the driving forces for the construction of films. Consequently, both synthetic polymers and biomaterials, which include proteins [10,11], polysaccharides [12,13], and DNA [14,15], are often used as components of LbL films. An advantage of LbL films is that the thickness of the film depends on the number of layers; thus, the thickness can be controlled at the nanometer level.

LbL films can be endowed with optical, electrical, chemical, and biological functions. For instance, LbL films containing fluorescent groups exhibit environment-dependent fluorescence [16]. On the other hand, LbL films modified with redox-active groups are used for the fabrication of electrochemical sensors [17]. Electrochemical enzyme sensors have been constructed by combining redox-active LbL films and enzymes, in which the LbL film serves as an electron transfer mediator between the enzyme and electrode [18]. Functional LbL films that decompose or deform in response to changes in external stimuli, such as pH [19], ionic strength [20], temperature [21], light irradiation [22], electric potential [23], and the concentrations of small molecules [24], have been also reported.

Recently, LbL films with selective binding sites have been developed using proteins and host-guest compounds. In this context, we have recently reviewed the synthesis and applications of LbL assemblies containing lectin proteins [25] and cyclodextrins [26]. LbL films containing selective binding sites are useful for biomedical applications, such as biosensors and controlled release.

**Figure 1:**
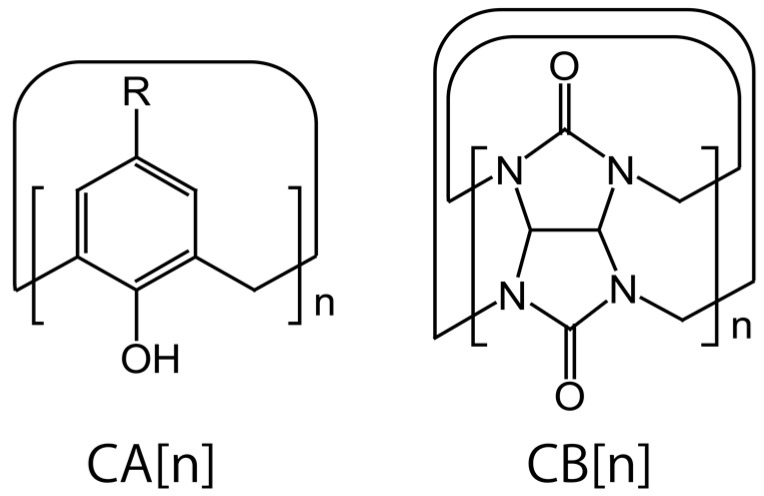
Chemical structures of CA[*n*] and CB[*n*].

This review provides an overview of LbL films containing calix[*n*]arenes (CA[*n*]s) and cucurbit[*n*]urils (CB[*n*]s) as selective binding sites. CA[*n*] is a class of organic macrocycles comprising phenolic moieties bridged by methylene units to form a hydrophobic cavity with a polar lower rim and a nonpolar upper rim (Figure 1). The letter [*n*] represents the number of phenolic moieties in the CA[*n*]s, among which CA[4], CA[6], and CA[8] have widely been studied because they are easy to prepare. CA[*n*] derivatives can form host-guest complexes with ions and molecules depending on the type of substituents on the lower and upper rims. Therefore, CA[*n*]s are utilized as components for the construction of molecular receptors and containers, ion transporters, catalysts, the stationary phase of chromatography, and so forth [27,28,29]. On the other hand, CB[*n*]s are products of a condensation reaction of glycoluril with formaldehyde [30,31,32]. CB[*n*]s are characterized by a hydrophobic cavity with two portals containing electron-rich carbonyl groups (Figure 1). Consequently, CB[*n*]s strongly bind to electron-deficient hydrophobic guests such as methyl viologen (MV) and cationic derivatives of adamantane and ferrocene [33,34]. Furthermore, CB[8] forms highly-stable ternary complexes with MV and electron-donating compounds such as naphthalene (the binding constant of the ternary complex, 1 × 10^12^ M^−2^) [35]. Thus, CA[*n*]s and CB[*n*]s form host-guest inclusion complexes with ions and molecules depending on the sizes and shapes. Therefore, CA[*n*]s and CB[*n*]s are useful for the construction of thin film devices with selective binding sites.

## 2. CA[*n*]-Containing LbL Films

Sulfonated CA[*n*]s (s-CA[*n*]s) were first employed by Swanson and coworkers for the fabrication of chemical sensors [36]. The group successfully prepared LbL films by the alternate deposition of sulfonated calix[6]arene (s-CA[6]) and poly(diallyldimethylammonium chloride) (PDDA) and characterized the films by means of surface acoustic wave (SAW) sensors and a scanning electron microscope. The thickness of a five-bilayer (PDDA/s-CA[6])_5_ film was estimated to be approximately 17 nm based on the SAW data. The s-CA[6]-containing LbL film-coated SAW sensors exhibit responses to the vapor of halogenated hydrocarbons, such as carbon tetrachloride, 1,1,1-trichloroethane, and chloroform at the ppm level. The results were rationalized based on the host-guest complexation of the volatile organic compounds (VOCs) and s-CA[6] in the film. Recently, VOCs sensors have been fabricated using optical fiber long-period gratings (LPGs) with s-CA[*n*]-containing LbL films [37]. Fiber optic sensors with LPGs have been widely used to measure VOCs as well as humidity and biomolecules [38,39,40]. LPG sensors coated with s-CA[*n*] films show a response to vapors of various VOCs, including chloroform, benzene, toluene, and acetone. Sensors coated with s-CA[4] film exhibit greater responses than those coated with s-CA[8] film, probably due to the complementary sizes of the VOCs and s-CA[4]’s cavities. The sensors showed different responses to two different commercial paint products, although they were unable to discriminate between the individual VOCs. Thus, CA[*n*]-containing LbL films are useful for the construction of VOC sensors.

CA[*n*]-containing LbL films can be prepared through hydrogen and covalent bonds. Covalently-linked LbL films containing CA[*n*] have been prepared by using cationic diazoresin (DR) and CA[*n*]s [41]. DR is known to react with substituents such as carboxylate and sulfonate groups to form covalent bonds under UV light irradiation [42]. The stability of LbL films composed of DR and CA[*n*]s is greatly enhanced by the formation of covalent bonds under irradiation. On the other hand, Cao and coworkers prepared single-component LbL films using CA[4] substituted with NH_3_^+^ and OH groups [43]. The LbL films were stabilized through hydrogen bonds between NH_3_^+^ and OH groups. The authors suggested that single-component LbL films have potential use for the construction of electrochemical sensors for Cu^2+^ ion. The same group used amphiphilic s-CA[*n*]s as molecular containers for immobilizing small molecules in LbL films [44]. To achieve this goal, s-CA[8] bearing C_12_ alkyl chains were alternately deposited with PDDA in the presence of organic dyes such as *p*-hydroxyazobenzene, methyl yellow, and Sudan III. The LbL films thus-prepared showed absorption bands associated with the dyes, suggesting that the dyes were incorporated into the cavity of s-CA[8] in the films. The results suggest the usefulness of s-CA[*n*]s as carriers for the immobilization of small molecules in LbL films.

Tieke and coworkers have systematically studied the permeability of CA[*n*]-containing LbL films deposited on the surfaces of porous polymer supports made of poly(acrylonitrile) and poly(ethylene terephthalate) [44,45,46,47,48,49]. s-CA[4], s-CA[6], and s-CA[8] were successfully built into LbL films by combining polycations, such as poly(vinyl amine) (PVA) and poly(allylamine hydrochloride). The deposition behavior of s-CA[*n*]s depends on the ring size of s-CA[*n*]s; a higher amount of s-CA[8] than s-CA[4] and s-CA[6] is deposited in the LbL film, depending on the number of the binding sites, that is, the sulfonate residues, in the s-CA[*n*]s.

**Figure 2:**
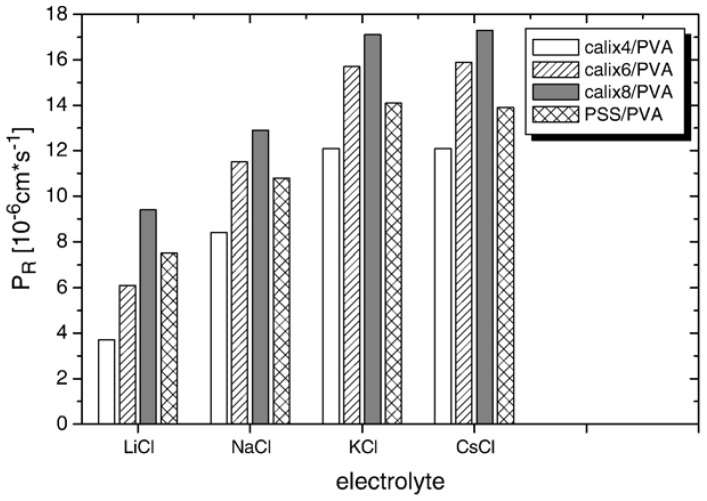
Permeation rates, *P*_R_, of alkaline metal chlorides across the (s-CA[*n*]/PVA)_60_ and (PSS/PVA)_60_ films. Reprinted with permission from [48]. Copyright 2008 Elsevier.

Ion permeation across 60-bilayered (s-CA[*n*]/PVA)_60_ films has been studied using a U-shaped two-compartment cell. The rate of ion permeation was measured by recording the conductivity of the solution in the receiving phase. All (s-CA[*n*]/PVA)_60_ films (*n* = 4, 6, and 8) exhibited higher permeability to monovalent alkali metal ions than to other ions. In addition, the permeation rates of divalent ions were higher than those of trivalent ions. It is plausible that multivalent ions are electrostatically rejected (i.e., Donnan rejection) by the positively-charged sites in films more strongly than monovalent ions. The permeation rates of the alkali metal ions tested increased as the ion sizes increased, showing that the effects of Donnan rejection are decreased by increasing the sizes of the ions or decreasing the charge density. In addition to size effects, the authors suggested that the complexation between CA[*n*]s and alkali metal ions, especially Li^+^ ion, is based on a significant dependence of the Li^+^ ion permeation rates on the sizes of the CA[*n*]s. The permeation rates of alkali metal ions across the LbL films were in the order of s-CA[8] > s-CA[6] > s-CA[4] (Figure 2), whereas divalent ions showed the highest permeation rates for s-CA[4]-containing films. These results also suggest the formation of complexes of divalent ions and s-CA[*n*]s in the films. The authors suggested that s-CA[*n*]-containing LbL films could potentially be used for the selective enrichment of rare earth metal ions because the permeation rates of Ln^3+^, La^3+^, Ce^3+^, Pr^3+^, and Sm^3+^ ions are extremely low.

**Figure 3:**
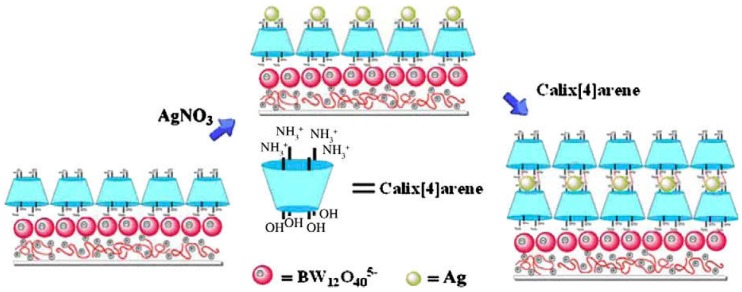
Synthesis of Ag nanoparticles in tetraamino-CA[4] film. Reprinted with permission from [50]. Copyright 2010 Elsevier.

It is anticipated that CA[*n*]-containing LbL films could be used as nano-reactors by taking advantage of the ability of CA[*n*]s to form inclusion complexes with ions and small molecules. In fact, Cao and coworkers have used LbL films consisting of tetraamino-CA[*n*]s as nano-reactors to synthesize silver (Ag) nanoparticles (Figure 3) [50]. Ag^+^ ions were selectively bound into the cavity of CA[*n*]s in the LbL film from the solution through π-cation interactions [51,52], followed by in situ reduction of Ag^+^ ions to Ag nanoparticles. Using this procedure, well-dispersed Ag nanoparticles with average diameters of 5–6 nm were prepared. The authors postulated that the *p*-aminophenol moieties in the CA[*n*]s were oxidized into *p*-iminobenzoquinone when reducing the Ag^+^ ions into metal nanoparticles. Monomolecular films of CA[*n*]s have likewise been used for in situ formation of Ag nanoparticles [53].

**Figure 4:**
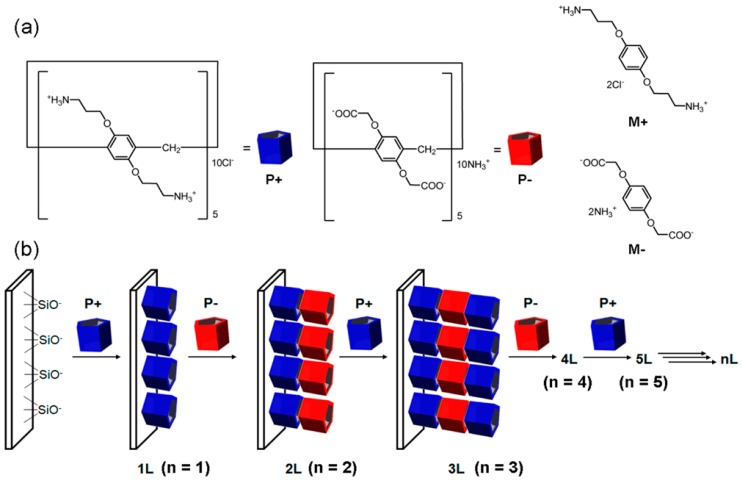
(**a**) Chemical structures of diamino-PA[5] and dicarboxyl-PA[5]; (**b**) LbL deposition of a multilayer film composed of diamino-PA[5] and dicarboxyl-PA[5]. Reprinted with permission from [56]. Copyright 2015 American Chemical Society.

Pillar-shaped macrocyclic compounds, pillar[*n*]arenes (PAs), have also been built into LbL films as components for the construction of molecular containers. PAs were first synthesized in 2008 by Ogoshi and coworkers as a novel class of host compounds [54,55]. Diamino-pillar[5]arene (diamino-PA[5]) and dicarboxyl-pillar[5]arene (dicarboxyl-PA[5]) (Figure 4) were alternately deposited on the surface of quartz plates to form LbL films through electrostatic affinity [56]. UV-VIS absorption spectroscopy revealed the successful preparation of PA[5] multilayer films. Interestingly, the PA[5] films showed selective binding to *p*-dinitrobenzene (*p*-DNB) over *m*- (*m*-DNB) and *o*-isomers (*o*-DNB). The results were rationalized based on the fact that the cavity size of PA[5] is approximately 5 Å, which is larger than the size of *p*-DNB (4.3 Å), whereas it is smaller than the cavities of *m*-DNB (5.9 Å) and *o*-DNB (5.9 Å). The loading of *p*-DNB onto the film increased as the number of PA5 layers increased. In addition, *p*-DNB did not bind to the films with a dicarboxyl-PA[5] outermost surface, although *p*-DNB was bound to LbL films terminated with positively-charged diamino-PA[5]. This is probably due to the negatively-polarized nature of *p*-DNB, which originates from an electron-withdrawing effect of the nitro groups. Recently, the same group has reported that the binding and release of *p*-DNB can be photochemically controlled by using azobenzene-capped PA[5] (azo-PA[5]) [57]. LbL films of azo-PA[5] bind *p*-DNB when azobenzene residues are in the trans form, whereas, upon UV light irradiation, the binding and/or release of *p*-DNB is blocked as a result of trans-to-cis isomerization of azobenzene group. Thus, the azobenzene group serves as photo-sensitive gate of the LbL films.

The loading and release of guest molecules into LbL films containing PA[6] has been studied using MV as the guest [58]. Covalently-linked LbL films were prepared by the alternate deposition of dicarboxyl-PA[6] complexed with MV and photosensitive polymer DR followed by UV light irradiation. The LbL film showed selective binding and release of MV several times repeatedly without significant deterioration. LbL films consisting of ammonium PA[5] and Au nanoparticles can be sued as plasmonic thin films for label-free detection of low molecular weight polyaromatic hydrocarbons (PAHs) in liquid and gas phases [59]. It is a merit of this film to bind PAHs without affinity to Au through host-guest complexation with PA[5].

## 3. CB[*n*]-Containing LbL Films

### 3.1. LbL Films on Flat Substrates

It may be possible to use CB[*n*]s as molecular linkers for the construction of LbL films composed of polymeric materials based on the fact that CB[*n*]s have been utilized as binders to prepare supramolecular assemblies such as hydrogels, micelles, and vesicles [60,61,62]. LbL films composed of CB[*n*]-coated Au nanoparticles (AuNPs) have been used to amplify the output signal of biosensors based on surface plasmon resonance (SPR) spectroscopy. SPR-based biosensors are useful tools for detecting biomolecules because the sensors can be operated without labeling the signaling molecules, such as fluorescence probes and enzymes. However, the intensity of the output signal is often too weak to detect low levels of substrates, such as antibodies and biomarkers. Consequently, a signal amplification protocol for SPR sensors is important for practical applications. Chen and coworkers deposited CB[7]-coated AuNPs/peptide-modified AuNPs multilayer films on the surface of an Au chip of an aSPR sensor [63]. For this purpose, a heptamer peptide consisting of a glycine pentamer with phenylalanine and cysteine end groups (NH_2_-Phe-Gly-Gly-Gly-Gly-Gly-Cys) was used. The AuNPs multilayer films were constructed through host-guest binding of the Phe moiety of the peptide into the CB[7] cavity. The SPR sensor was used to determine caspase-3 enzyme. The output signal of the sensor without LbL film was 0.03 degrees for 10^5^ pg·mL^−1^ caspase-3, which was not sensitive enough to detect the substrate. On the other hand, the intensity of the signal was enhanced 20-fold through the deposition of four-layered AuNPs on the Au chip. The amplified SPR sensor exhibits a linear detection range of 10–10^3^ fg·mL^−1^ with a lower detection limit of 2.2 fg·mL^−1^. Thus, CB[*n*]-based LbL films have been successfully used to amplify the output signals of SPR biosensors.

**Figure 5:**
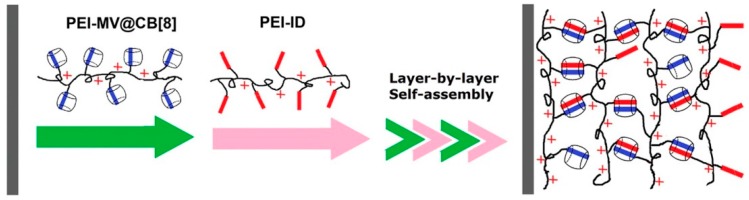
The preparation of (PEI-MV@CB[8]/PEI-ID)*n* LbL film. Reprinted with permission from [65]. Copyright 3013 American Chemical Society.

**Figure 6:**
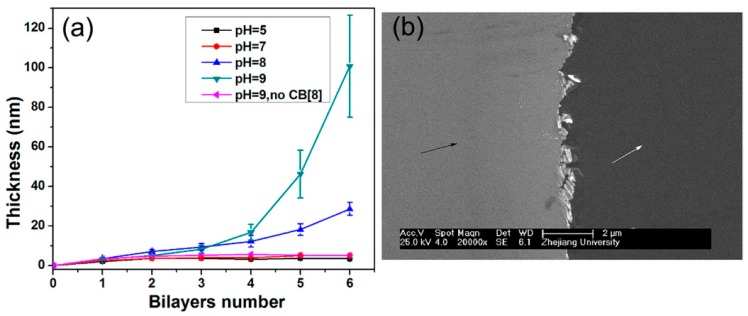
(**a**) The thickness of (PEI-MV@CB[8]/PEI-ID)*n* film as a function of the number of bilayers prepared at different pH levels; (**b**) scanning electron microscopy image of (PEI-MV@CB[8]/PEI-ID)6 film prepared at pH 9 (the left side of the image marked by the black arrow indicates the surface of the LbL film). Reprinted with permission from [65]. Copyright 2013 American Chemical Society.

It is known that CB[8] forms a stable 1:1:1 ternary complex with electron-rich and electron-deficient molecules, in which the ternary complex is stabilized through host-guest complexation, as well as charge transfer interactions [64]. In fact, LbL films comprising PEIs covalently modified with electron-rich indole (PEI-ID) and electron-deficient methyl viologen moieties (PEI-MV) have been prepared using CB[8] as the binder [65]. A solid substrate was immersed in a solution of PEI-MV and CB[8] to deposit the first layer of PEI-MV complexed with CB[8] (PEI-MV@CB[8]), followed by the deposition of PEI-ID through the formation of ternary complexes of In and MV moieties in the cavity of CB[8]. Figure 5 shows the effect of pH on the formation of the LbL films. The LbL film can be prepared at pH 8 and 9, but does not form successfully when the pH is less than 7. This is probably due to the lower stability of the ternary complex as well as an electrostatic repulsion between the positive charges of the PEI polymers at lower pH values. The surface of the LbL film is rather smooth, as shown by the AFM image in Figure 6. Interestingly, the six-bilayar LbL film prepared at pH 9.0 was stable in solutions of pH 4, 7.4, and 9 at 37 °C, despite unsuccessful deposition of the film at pH 4 and 7.4. On the other hand, the LbL film decomposed instantly upon adding 1 mg·mL^−1^ adamantane (Ad) as a result of the competitive binding of Ad to the cavity of the CB[8]. CB[8]-based LbL films are more stable than similar LbL films linked with cyclodextrin [66,67] due to the ternary complexes possessing a higher binding constant (approximately 1 × 10^12^ M^−2^) [64].

A supramolecular pseudo-polycation composed of naphthalene-bearing dextran (NpD), MV, and CB[8] has been utilized as a cationic component for the construction of LbL films with poly(acrylic acid) (PAA) through electrostatic affinity [68]. The (PAA/NpD + MV + CB[8])*_n_* LbL films decomposed in the presence of Ad or Na_2_S_2_O_4_ as a result of the competitive binding of Ad to CB[8] or a reduction of MV by Na_2_S_2_O_4_. It was also possible to use (PAA/NpD + MV + CB[8])*_n_* LbL films as sacrificial layers for preparing free-standing LbL films. The (PAA/NpD + MV + CB[8])*_n_* films were further coated with another LbL film consisting of PSS and PDDA, and the composite LbL film was exposed to Ad or Na_2_S_2_O_4_ solution to release the PSS/PDDA film from the substrate. The critical thickness of the (PAA/NpD + MV + CB[8])*_n_* film to enable the release of free-standing PSS/PDDA film is *n* = 5 (or approximately 160 nm).

**Figure 7:**
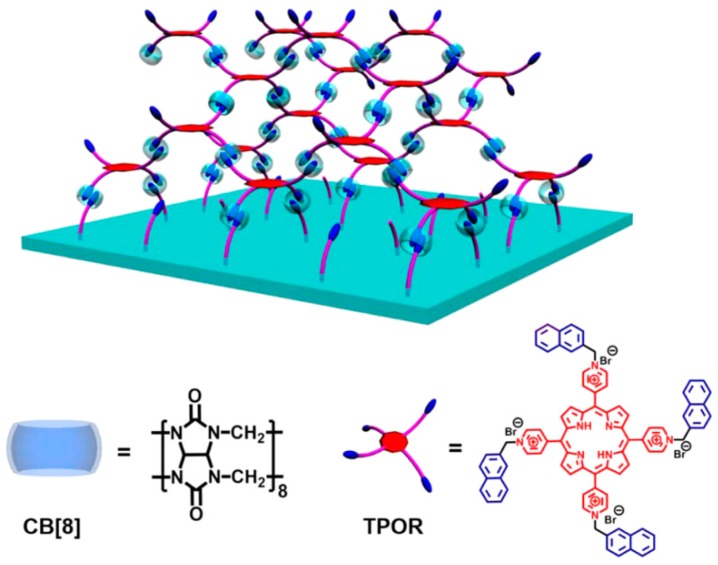
LbL film composed of CB[8] and TPOR. Reprinted with permission from [69]. Copyright 2014 American Chemical Society.

In other examples, CB[8] has been used to link small molecules to each other in LbL films. Zhang and coworkers have prepared LbL films by the alternate deposition of CB[8] and multi-armed compounds such as naphthalene-substituted tetrapyridyl(porphyrin) (TPOR) [69] (Figure 7) or naphthalene-substituted tris(imidazole) (BNTI) [70]. TPOR and BNTI are linked by CB[8] in the films by forming dimers of the naphthalene units in the cavity of CB[8]. The TPOR/CB[8] LbL films catalyze a photochemical oxidation reaction of 1,5-dihydroxynaphthalene into 5-hydroxy-1,4-naphthoquinone under UV light irradiation. The TPOR/CB[8] film can also be used for the photo-catalytic oxidation of hydroquinone, 1-naphthole, and 1,3,5-trihydroxybenzene. CB[8] can also be used as a linker for the construction of protein-based LbL films [71]. The alternate deposition of CB[8] and proteins such as hemoglobin (Hb) and catalase (CAT) afforded multilayered protein films. A quartz crystal microbalance study revealed that the thickness of the Hb/CB[8] bilayer is approximately 9.1 nm, in accord with the sum of the dimensions of Hb (6.5 × 7.8 × 10.9 nm^3^) and CB[8] (0.9 × 1.6 × 1.6 nm^3^) molecules, suggesting that Hb and CB[8] form monomolecular layers in the LbL film. The presence of surface-exposed aromatic amino acids in the proteins may be responsible for the successful deposition of CB[8]/protein LbL films. Thus, CB[8] is a promising synthetic glue, similar to binding proteins, such as avidin and lectin [72,73], for the construction of protein nano-assemblies.

**Figure 8:**
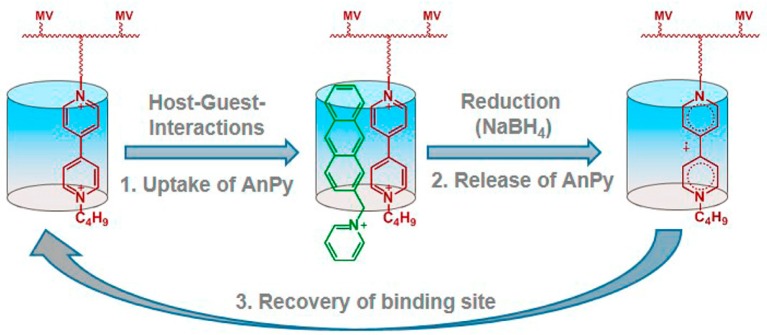
Uptake of AnPy in the CB[8]/viologen LbL film and its release triggered by NaBH_4_ reduction. Reprinted with permission from [74]. Copyright 2015 American Chemical Society.

It is anticipated that CB[*n*]-containing LbL films will be suitable for use as scaffolds for the binding and release of small molecules by taking advantage of the binding ability of CB[*n*]s. In this context, LbL films have been prepared by the alternate deposition of PAA and a cationic polymer bearing viologen side chains complexed with CB[8] [74]. The LbL films thus prepared were able to bind anthracene-substituted pyridinium bromide (AnPy), which formed charge-transfer complexes with the viologen moiety in the cavity of CB[8] in the film. On the other hand, AnPy was released from the film by reducing the viologen moieties into the radicals with NaBH_4_ because of the loss of affinity of AnPy to the reduced viologen moiety (Figure 8). The durability of LbL films via photochemical cross-linking of the film components can be enhanced using an azide-bearing anionic polymer in place of PAA [75].

Photosensitive chemical systems in which the structures and functions can be regulated by light irradiation have been widely studied [76,77,78,79]. In this regard, photo-sensitive LbL films have been developed using azobenzene-modified polycations and PAA to photochemically control the binding and release of guest molecules [80]. The LbL films were deposited from the solutions of PAA and azobenzene-modified polycation in the presence of CB[8] to accommodate the azobenzene residues into the CB[8] cavity in the film. The azobenzene/CB[8] complexes further accommodated MV from the solution to form a MV/azobenzene/CB[8] ternary complex in the film. The MV was released from the film upon UV light irradiation owing to the decomposition of the ternary complexes, which in turn resulted in trans-to-cis isomerization of the azobenzene residues. MV could be bound into the LbL film and released 10 times repeatedly without significant degradation of the film.

### 3.2. LbL Films Coated on the Surface of Colloidal Particles and Nanopores

**Figure 9:**
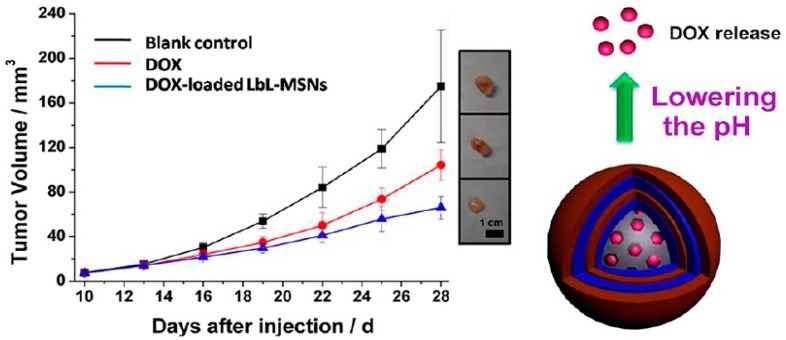
(**Left**) Increase in tumor volume in model mice untreated, treated with DOX, or treated with DOX-loaded MSNs. (**Right**) A schematic illustration of the release of DOX from DOX-loaded MSNs triggered by pH changes. Reprinted with permission from [84]. Copyright 2014 American Chemical Society.

CB[*n*]-containing LbL films have been coated on mesoporous silica nanoparticles (MSNs) for the development of drug delivery systems. MSNs are currently widely used as carriers for controlled release because of their excellent properties including easy preparation, high stability and rigidity, high surface area, and biocompatibility [81,82,83]. Gao and Yang group have synthesized two types of MSNs (average diameter, 190 nm) with pore sizes of 2.7 and 5.5 nm (MSN-1 and MSN-2, respectively) for anticancer drug delivery [84]. The MSNs were first loaded with the anticancer drug doxorubicin (DOX) by immersing the MSNs in DOX solution followed by coating LbL films composed of a poly(methacrylate) derivative bearing ethylenediamine side chains and CB[7]. The LbL films were stabilized through a cross-linking of the poly(methacrylate) chains by CB[7] through ion-dipole interactions. The DOX-loaded MSNs were exposed to aqueous solutions of pH 2.0, 5.0, and 7.4 to evaluate the release of DOX. The release of DOX was accelerated at pH 2.0 and 5.0, while it was very slow at pH 7.4. The results were rationalized based on the decomposition of LbL films at acidic pH levels, which originated from the competitive binding of H^+^ ions with the ethylenediamine-bearing poly(methacrylate) to CB[7]. It is known that the pH value of endosomes and lysosomes in tumor cells is lower (pH 5.0–5.5) than that of normal tissues. Therefore, the anti-cancer activity of DOX-loaded MSNs was tested in vivo using a mouse model containing HeLa cancer cells, in which the growth of the tumors was inhibited by 63% in mice administered with DOX-loaded MSNs compared to the untreated group (Figure 9).

CB[7] LbL film-coated MSNs loaded with tetraphenylethylene carboxylic acid (TPE-COOH) and antibiotic drug amoxicillin (AMO) have been shown to be effective for the detection and elimination of bacteria [85]. The fluorescence intensity at 480 nm originating from the TPE-COOH in the MSNs decreased upon exposure to bacteria such as *Escherichia coli* and *Staphylococcus aureus* because of the loss of aggregation-induced emission of the TPE-COOH. The detection limit was 2.5 × 10^6^ CFU·mL^−1^ for *E. coli* and 5 × 10^6^ CFU·mL^−1^ for *S. aureus* (CFU, colony forming unit). In addition, AMO was released from the MSNs in the presence of adamantaneamine owing to the decomposition of LbL films, thus providing antibacterial activity. Hence, the AMO/TPE-COOH-loaded MSNs are useful both for both the elimination and detection of bacteria.

Interestingly, a protocol for the one-step preparation of polymer microcapsules has been developed using CB[8] in microfluidic devices [86,87,88,89,90,91]. Different techniques and materials can be used for the preparation of polymer microcapsules, such as polymersomes [92], self-assembled vesicles [93], and LbL films [94,95]. Among them, LbL film-based microcapsules are currently widely used for the development of micro-containers for drug delivery because they are simple to prepare under mild conditions. In fact, LbL microcapsules can be fabricated by placing LbL film coating on template microparticles that contain cargo molecules such as drugs and proteins, followed by dissolution of the template to form hollow capsules leaving the cargo molecules in the cavity. A drawback of the LbL film-based microcapsules comes from the limited efficiency of encapsulation due to leakage of the cargo molecules during the film-coating process and dissolution of the template particles. In contrast, Abell and coworkers have developed a novel protocol for the preparation of polymer microcapsules capable of encapsulating sufficient amounts of cargo molecules [86,87,88,89,90,91]. The microcapsules are prepared using MV-modified Au nanoparticles and naphthalene polymers bridged by CB[8]. In this protocol, aqueous microdroplets containing the Au nanoparticle, naphthalene polymer, and CB[8] are produced in microfluidic devices using an oil phase. Polymer thin films form at the oil/water interface and then stable microcapsules are isolated within minutes after evaporation of the oil phase and dehydration. The diameter of the microcapsules can be tuned from 10 to 24 µm by changing the flow rates of the aqueous and oil phases in the microfluidic device. The polymer microcapsules thus prepared may be promising tools in the development of devices for controlled release, similarly to LbL film-based microcapsules.

**Figure 10:**
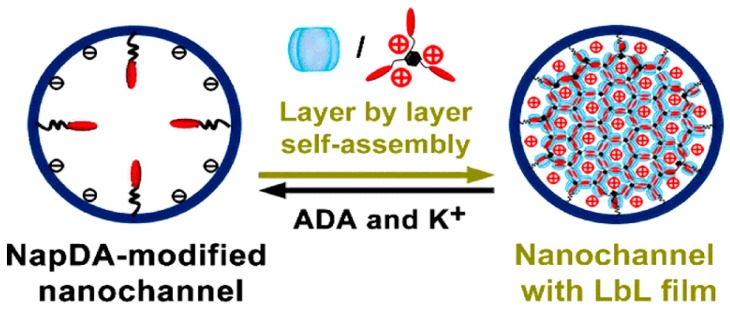
NapDA-coated nanochannel modified with CB[8]/BTNI LbL films. Reprinted with permission from [98]. Copyright 2016 American Chemical Society.

Nanomaterials with nanopores and nanochannels have attracted much attention in relation to the significant roles of ion channels in biological cells [96,97]. To mimic biological ion channels, a polyethylene terephthalate membrane was etched in NaOH solution to create conical nanochannels with diameters of 186 ± 28 nm on the larger side and 31 ± 12 nm on the smaller side and the inner surfaces of the nanopores were coated with LbL films [98]. The surfaces of the nanopores were first covalently modified with a naphthalene derivative (NapDA), through which CB[8] was anchored, followed by binding of BTNI via the formation of NapDA/CB[8]/BTNI ternary complexes. The ternary complex could be decomposed by adding Ad or K^+^ ion to restore the NapDA-modified nanopores. The ion current of the nanopore membrane measured in 0.1 M NaCl solution depended on the type of surface architecture. The rectification ratio, defined by the ratio of ion current observed at –0.2 V to the current at +0.2 V, changed reversibly. It was further possible to construct LbL layers composed of CB[8] and BTNI on the nanopore surface for ion gating (Figure 10). The ion current of the LbL film-coated nanopore membranes recorded at –0.2 V was 20 nA for the NapDA-modified nanopore and 1 nA for five-bilayer LbL film-coated nanopore. Thus, CB[8] is useful for the construction of LbL film-modified nanopore membranes.

## 4. Conclusions

This review has demonstrated that CA[*n*]s and CB[*n*]s can be successfully built into LbL assemblies and serve as selective binding sites for ions and molecules. CA[*n*]-containing LbL films are useful for the construction of chemical sensors for VOCs and perm-selective membranes for metal ions. A merit of CA[*n*]s comes from the simple preparation of water-soluble derivatives such as s-CA[*n*]s, in contrast to the limited solubility of unmodified CA[*n*]s. On the other hand, CB[*n*]-containing LbL films and microcapsules are useful for the construction of molecular containers and controlled release systems owing to their extremely strong affinity for positively-charged organic guests such as MV. The selective binding properties of CA[*n*]s and CB[*n*]s can be further enhanced by combining the host molecules with nanomaterials such as MSNs and nanopore membranes. Thus, CA[*n*]s- and CB[*n*]s-modified LbL assemblies may be promising tools for biomedical applications.

## References

[1] Decher G., Hong J.D., Schmitt J. (1992). Buildup of ultrathin multilayer films by a self-assembly process: III. Consecutively alternating adsorption of anionic and cationic polyelectrolytes on charged surfaces. Thin Solid Films.

[2] Lvov Y., Decher G., Möhwald H. (1993). Assembly, structural characterization, and thermal behavior of layer-by-layer deposited ultrathin films of poly(vinyl sulfate) and poly(allylamine). Langmuir.

[3] Tomita S., Sato K., Anzai J. (2008). Layer-by-layer assembled thin films composed of carboxyl-terminated poly(amidoamine) dendrimer as a pH-sensitive nano-device. J. Colloid Interface Sci..

[4] Kharlampieva E., Kozlovskaya V., Sukhishvili S.A. (2009). Layer-by-layer hydrogen-bonded polymer films: From fundamentals to applications. Adv. Mater..

[5] Akiba U., Anzai J. (2017). Cyclodextrin-containing layer-by-layer films and microcapsules: Synthesis and applications. AIMS Mater. Sci..

[6] Shimazaki Y., Mitsuishi M., Ito S., Yamamoto M. (1997). Preparation of the layer-by-layer deposited ultrathin film based on the charge-transfer interaction. Langmuir.

[7] Chen G., Wang X., Liu A., Qian D. (2009). Layer-by-layer assembly of poly(*p*-xylyleneviologen)-DNA multilayers and their electrochemical properties. Mater. Sci. Eng. C.

[8] Zayas-Gonzalez Y.M., Lynn D.M. (2016). Degradable amine-reactive coatings fabricated by the covalent layer-by-layer assembly of poly(2-vinyl-4,4-dimethylazlactone) with degradable polyamine building blocks. Biomacromolecules.

[9] Anzai J., Kobayashi Y., Suzuki Y., Takeshita H., Chen Q., Osa T., Hoshi T., Du X. (1998). Enzyme sensors prepared by layer-by-layer deposition of enzymes on a platinum electrode through avidin-biotin interaction. Sens. Actuators B.

[10] Yoshida K., Hasebe Y., Takahashi S., Sato K., Anzai J. (2014). Layer-by-layer deposited nano- and micro-assemblies for insulin delivery: A review. Mater. Sci. Eng. C.

[11] Onda M., Ariga K., Kunitake T. (1999). Activity and stability of glucose oxidase in molecular films assembled alternately with polyions. J. Biosci. Bioeng..

[12] Wang B., Anzai J. (2007). Redox reactions of ferricyanide ions in layer-by-layer deposited polysaccharide films: A significant effect of the type of polycation in the films. Langmuir.

[13] Crouzier T., Boudou T., Picart C. (2010). Polysaccharide-based polyelectrolyte multilayers. Curr. Opin. Colloid Interface Sci..

[14] Sato H., Anzai J. (2006). Preparation of layer-by-layer thin films composed of DNA and ferrocene-bearing poly(amine)s and their redox properties. Biomacromolecules.

[15] Johnston A.P.R., Caruso F. (2008). Stabilization of DNA multilayer films through oligonucleotide crosslinking. Small.

[16] Li Y., Huang H., Li Y., Su X. (2013). Highly sensitive fluorescent sensor for mercury (II) ion based on layer-by-layer self-assembled films fabricated with water-soluble fluorescent conjugated polymer. Sens. Actuators B.

[17] Sato K., Takahashi S., Anzai J. (2012). Layer-by-layer thin films and microcapsules for biosensors and controlled release. Anal. Sci..

[18] Takahashi S., Anzai J. (2013). Recent progress in ferrocene-modified thin films and nanoparticles for biosensors. Materials.

[19] Yoshida K., Sato K., Anzai J. (2010). Layer-by-layer polyelectrolyte films containing insulin for pH-triggered release. J. Mater. Chem..

[20] Pál E., Sebök D., Hornok V., Dékány I. (2009). Structure, optical, and adsorption properties of ZnO_2_/poly(acrylic acid) hybrid thin porous films prepared by ionic strength controlled layer-by-layer method. J. Colloid Interface Sci..

[21] Liang X., Kozlovskaya V., Chen Y., Zavgorodnya O., Kharlampieva E. (2012). Thermosensitive multilayer hydrogels of poly(*N*-vinylcaprolactam) as nanothin films and shaped capsules. Chem. Mater..

[22] Wang X., Chen W., Zhang J., Li C., Zhuo R., Zhang X. (2011). Design of a photoswitchable hollow microcapsular drug delivery system by using a supramolecular drug-loading approach. J. Phys. Chem. B.

[23] Sato K., Kodama D., Naka Y., Anzai J. (2006). Electrochemically induced disintegration of layer-by-layer-assembled thin films composed of 2-iminobiotin-labeled poly(ethyleneimine) and avidin. Biomacromolecules.

[24] Watahiki R., Sato K., Suwa K., Niina S., Egawa Y., Seki T., Anzai J. (2014). Multilayer films composed of phenylboronic acid-modified dendrimers sensitive to glucose under physiological conditions. J. Mater. Chem. B.

[25] Wang B., Anzai J. (2015). Recent progress in lectin-based biosensors. Materials.

[26] Sato K., Suzuki I., Anzai J. (2003). Preparation of polyelectrolyte-layered assemblies containing cyclodextrin and their binding properties. Langmuir.

[27] Sansone F., Baldini L., Casnati A., Ungaro R. (2010). Calixarenes: From biomimetic receptors to multivalent ligands for biomolecular recognition. New J. Chem..

[28] Gezici O., Bayrakci M. (2015). Calixarene-engineered surfaces and separation science. J. Incl. Phenom. Macrocycl. Chem..

[29] Ovsyannikov A., Solovieva S., Antipin I., Ferlay S. (2017). Coordination polymers based on calixarene derivatives: Structures and properties. Coord. Chem. Rev..

[30] Walker S., Oun R., McInnes F.J., Wheate N.J. (2011). The potential of cucurbit[*n*]urils in drug delivery. Isr. J. Chem..

[31] Masson E., Ling X., Joseph R., Kyeremeh-Mensah L., Lu X. (2012). Cucurbituril chemistry: A tale of supramolecular success. RSC Adv..

[32] Gürbüz S., Idris M., Tuncel D. (2015). Cucurbituril-based supramolecular engineered nanostructured materials. Org. Biomol. Chem..

[33] Das D., Scherman O.A. (2011). Cucurbituril: At the interface of small molecule host-guest chemistry and dynamic aggregates. Isr. J. Chem..

[34] Kaifer A.E. (2017). Electrochemical properties of cucurbit[7]uril complexes of ferrocenyl derivatives. Inorg. Chim. Acta.

[35] Jeon Y.J., Bharadwaj P.K., Choi S.W., Lee J.W., Kim K. (2002). Supramolecular amphiphiles: Spontaneous formation of vesicles triggered by formation of a charge-transfer complex in a host. Angew. Chem. Int. Ed..

[36] Yang X., Johnson S., Shi J., Holesinger T., Swanson B. (1997). Polyelectrolyte and molecular host ion self-assembly to multilayer thin films: An approach to thin film chemical sensors. Sens. Actuators B.

[37] Hromadka J., Korposh S., Partridge M., James S.W., Davis F., Crump D., Tatam R.P. (2017). Volatile organic compounds sensing using optical fibre long period grating with mesoporous nano-scale coating. Sensors.

[38] Korposh S., Selyanchyn R., Yasukochi W., Lee S.W., James S.W., Tatam R.P. (2012). Optical fibre long period grating with a nanoporous coating formed from silica nanoparticles for ammonia sensing in water. Mater. Chem. Phys..

[39] Korposh S., Wang T., James S., Tatam R., Lee S.W. (2012). Pronounced aromatic carboxylic detection using a layer-by-layer mesoporous coating on optical fibre long period grating. Sens. Actuators B.

[40] Hromadka J., Tokay B., James S., Tatam R.P., Korposh S. (2015). Optical fibre long period grating gas sensor modified with metal organic framework thin films. Sens. Actuators B.

[41] Yang Z., Cao W. (2003). Covalently attached multilayer ultra-thin films from diazoresin and calixarenes. Chin. J. Polym. Sci..

[42] Egawa Y., Hayashida R., Anzai J. (2007). Covalently cross-linked multilayer thin films composed of diazoresin and brilliant yellow for an optical pH sensor. Polymer.

[43] Gao S., Yuan D., Lu J., Li T., Cao R. (2007). Multilayer films of single-component and charged tetraaminocalix[4]arenes based on hydrogen bonding. Chem. Commun..

[44] Zhang Y., Cao W. (2001). Self-assembly of small molecules: An approach combining electrostatic self-assembly technology with host-guest chemistry. New J. Chem..

[45] Toutianoush A., Schnepf J., El-Hashani A., Tieke B. (2005). Selective ion transport and complexation in layer-by-layer assemblies of *p*-sulfonato-calix[*n*]arenes and cationic polyelectrolytes. Adv. Funct. Mater..

[46] Toutianoush A., El-Hashani A., Schnepf J., Tieke B. (2005). Multilayer membranes of *p*-sulfonato-calix[8]arene and polyvinylamine and their use for selective enrichment of rare earth metal ions. Appl. Surf. Sci..

[47] El-Hashani A., Tieke B. (2006). Electrostatic layer-by-layer assembly of ultrathin films containing hexacyclen and *p*-sulfonato-calix[*n*]arene macrocycles. J. Nanosci. Nanotechnol..

[48] Tieke B., El-Hashani A., Toutianoush A., Fendt A. (2008). Multilayered films based on macrocyclic polyamines, calixarenes and cyclodextrins and transport properties of the corresponding membranes. Thin Solid Films.

[49] Tieke B., Toutianoush A., Jin W. (2005). Selective transport of ions and molecules across layer-by-layer assembled membranes of polyelectrolytes, *p*-sulfonato-calix[*n*]arenes and Prussian blue-type complex salts. Adv. Colloid Interface Sci..

[50] Gao S., Yuan D., Lü J., Cao R. (2010). In situ synthesis of Ag nanoparticles in aminocalix[4]arene multilayers. J. Colloid Interface Sci..

[51] Petrella A.J., Raston C.L. (2004). Calixarenes as platforms for the construction of multimetallic complexes. J. Organomet. Chem..

[52] Csokai V., Grün A., Balázs B., Simon A., Tóth G., Bitter I. (2006). Functionalized thiacalix- and calix[4]arene-based Ag^+^ ionophores: Synthesis and comparative NMR study. Tetrahedron.

[53] Ruitao Z., Shileng T., Srinivasan M.P. (2014). In situ formation of silver nanoparticle layer by supramolecule-directed assembly. Thin Solid Films.

[54] Ogoshi T., Kanai S., Fujinami S., Yamagishi T., Nakamoto Y. (2008). para-Bridged symmetrical pillar[5]arenes: Their Lewis acid catalyzed synthesis and host-guest property. J. Am. Chem. Soc..

[55] Ogoshi T., Yamagishi T. (2014). Pillar[5]- and pillar[6]arene-based supramolecular assemblies built by using their cavity-size-dependent host-guest interactions. Chem. Commun..

[56] Ogoshi T., Takashima S., Yamagishi T. (2015). Molecular recognition with microporous multilayer films prepared by layer-by-layer assembly of pillar[5]arenes. J. Am. Chem. Soc..

[57] Ogoshi T., Takashima S., Yamagishi T. (2018). Photocontrolled reversible guest uptake, storage, and release by azobenzene-modified microporous multilayer films of pillar[5]arenes. J. Am. Chem. Soc..

[58] Yuan B., Xu J.F., Sun C.L., Nicolas H., Schonhöff M., Yang Q.Z., Zhang X. (2016). Pillar[6]arene containing multilayer films: Reversible uptake and release of guest molecules with methyl viologen moieties. ACS Appl. Mater. Interfaces.

[59] Montes-García V., Gómez-González B., Martinez-Solís D., Taboada J.M., Jiménez-Otero N., Uña-Álvarez J., Obelleiro F., García-Río L., Pérez-Juste J., Pastoriza-Santos I. (2017). Pillar[5]arene-based supramolecular plasmonic thin films for label-free, quantitative and multiplex SERS detection. ACS Appl. Mater. Interfaces.

[60] Apple E.A., Biedermann F., Rauwald U., Jones S.T., Zayed J.M., Scherman O.A. (2010). Supramolecular cross-linked networks via host-guest complexation with cucurbit[8]uril. J. Am. Chem. Soc..

[61] Chen C., Li D., Wang H., Zhao J., Ji J. (2013). Fabrication of dual-responsive micelles based on the supramolecular interaction of cucurbit[8]uril. Polym. Chem..

[62] Kushwaha S., Maity A., Gangopadhyay M., Ravindranathan S., Rajamohanan P.R., Das A. (2017). Cucurbit[7]uril induced formation of FRET-enhanced unilamellar lipid vesicles. Langmuir.

[63] Gao Y., Zou F., Wu B., Wang X., Zhang J., Koh K., Chen H. (2016). CB[7]-mediated signal amplification approach for sensitive surface plasmon resonance spectroscopy. Biosens. Bioelectron..

[64] Lagona J., Mukhopadhyay P., Chakrabarti S., Isaacs L., Lu X. (2005). The cucurbit[*n*]uril family. Angew. Chem. Int. Ed..

[65] Li D., Ren K., Chang H., Wang H., Wang J., Chen C., Ji J. (2013). Cucurbit[8]uril supramolecular assembly for positively charged ultrathin films as nanocontainers. Langmuir.

[66] Suzuki I., Egawa Y., Mizukawa Y., Hoshi T., Anzai J. (2002). Construction of positively-charged layered assemblies assisted by cyclodextrin complexation. Chem. Commun..

[67] Van den Heyden A., Wilczewski M., Labbé P., Auzléy R. (2006). Multilayer films based on host-guest interactions between biocompatible polymers. Chem. Commun..

[68] Li D., Chen X., Ren K., Ji J. (2015). Cucurbit[8]uril-based stimuli-responsive films as a sacrificial layer for preparation of free-standing thin films. Chem. Commun..

[69] Yuan B., Yang H., Wang Z., Zhang X. (2014). Interfacial fabrication of functional supramolecular polymeric networks for photocatalysis. Langmuir.

[70] Yang H., Ma Z., Yuan B., Wang Z., Zhang X. (2014). Supramolecular polymerization at the interface: Layer-by-layer assembly driven by host-enhanced π–π interaction. Chem. Commun..

[71] Yang H., An Q., Zhu W., Li W., Jiang Y., Cui J., Zhang X., Li G. (2012). A new strategy for effective construction of protein stacks by using cucurbit[8]uril as a glue molecule. Chem. Commun..

[72] Inoue H., Anzai J. (2005). Stimuli-sensitive thin films prepared by a layer-by-layer deposition of 2-iminobiotin-labeled poly(ehyleneimine) and avidin. Langmuir.

[73] Hoshi T., Akase S., Anzai J. (2002). Preparation of multilayer thin films containing avidin through sugar-lectin interactions and their binding properties. Langmuir.

[74] Nicolas H., Yuan B., Zhang J., Zhang X., Schönfoff M. (2015). Cucurbit[8]uril as nanocontainer in a polyelectrolyte multilayer film: A quantitative and kinetic study of guest uptake. Langmuir.

[75] Zhang J., Liu Y., Yuan B., Wang Z., Schönfoff M., Zhang X. (2012). Multilayer films with nanocontainers: Redox-controlled reversible encapsulation of guest molecules. Chem. Eur. J..

[76] Anzai J., Ueno A., Osa T. (1984). High and rapid response in photo-induced potential changes across a poly(vinyl chloride)/spirobenzopyran membrane. J. Chem. Soc. Chem. Commun..

[77] Anzai J., Osa T. (1994). Photosensitive artificial membranes based on azobenzene and spirobenzopyran derivatives. Tetrahedron.

[78] Goulet-Hanssens A., Sun K.L.W., Kennedy T.E., Barrett C.J. (2012). Photoreversible surfaces to regulate cell adhesion. Biomacromolecules.

[79] Akiba U., Minaki D., Anzai J. (2017). Photosensitive layer-by-layer assemblies containing azobenzene groups: Synthesis and biomedical applications. Polymers.

[80] Nicolas H., Yuan B., Zhang J., Zhang X., Schönfoff M. (2016). Cucurbit[8]uril-containing multilayer films for the photocontrolled binding and release of a guest molecule. Langmuir.

[81] Vallet-Regi M., del Real A.R.R.P., Perez-Pariente J. (2001). A new property of MCM-41: Drug delivery system. Chem. Mater..

[82] Wen J., Yang K., Liu F., Li H., Xu Y., Sun S. (2017). Diverse gatekeepers for mesoporous silica nanoparticle based drug delivery systems. Chem. Soc. Rev..

[83] Florek J., Caillard R., Kleitz F. (2017). Evaluation of mesoporous silica nanoparticles for oral drug deliver—Current status and perspective of MSNs drug carriers. Nanoscale.

[84] Li Q., Sun Y., Sun Y., Wen J., Zhou Y., Bing Q., Isaacs L.D., Jin Y., Gao H., Yang Y. (2014). Mesoporous silica nanoparticles coated by layer-by-layer self-assembly using cucurbit[7]uril for in vitro and in vivo anticancer drug release. Chem. Mater..

[85] Li Q., Wu Y., Lu H., Wu X., Chen S., Song N., Yang Y., Gao H. (2017). Construction of supramolecular nanoassembly for responsive bacterial elimination and effective bacterial detection. ACS Appl. Mater. Interfaces.

[86] Zhang J., Coulston R.J., Jones S.T., Geng J., Scherman O.A., Abell C. (2012). One-step fabrication of supramolecular microcapsules from microfluidic droplets. Science.

[87] Zheng Y., Yu Z., Parker R.M., Wu Y., Abell C., Scherman O.A. (2014). Interfacial assembly of dendritic microcapsules with host-guest chemistry. Nat. Commun..

[88] Yu Z., Zhang J., Coulston R.J., Parker R.M., Biedermann F., Liu X., Scherman O.A., Abell C. (2015). Supramolecular hydrogel microcapsules via cucurbit[8]uril host-guest interactions with triggered and UV-controlled molecular permeability. Chem. Sci..

[89] Parker R.M., Zhang J., Yu Z., Coulston R.J., Smith C.A., Salmon A.R., Yu Z., Scherman O.A., Abell C. (2015). Electrostatically directed self-assembly of ultrathin supramolecular polymer microcapsules. Adv. Funct. Mater..

[90] Yu Z., Lan Y., Parker R.M., Zhang W., Deng X., Scherman O.A., Abell C. (2016). Dual-responsive supramolecular colloidal microcapsules from cucurbit[8]uril molecular recognition in microfluidic droplets. Polym. Chem..

[91] Liu J., Lan Y., Yu Z., Tan C.S.Y., Parker R.M., Abell C., Scherman O.A. (2017). Cucurbit[*n*]uril-based microcapsules self-assembled within microfluidic droplets: A versatile approach for supramolecular architectures and materials. Acc. Chem. Res..

[92] Kim S., Nam J., Kim J.W., Kim D., Han S., Weitz D.A. (2013). Formation of polymersomes with double bilayers templated by quadruple emulsions. Lab Chip.

[93] Richter A.G., Dergunov S.A., Kim M.D., Shmakov S.N., Pingali S.V., Urban V.S., Liu Y., Pinkhassik E. (2017). Unraveling the single-nanometer thickness of shells of vesicle-templated polymer nanocapsules. J. Phys. Chem. Lett..

[94] Donath E., Sukhorukov G.B., Caruso F., Davis S.A., Möhwald H. (1998). Novel hollow polymer shells by colloid-templated assembly of polyelectrolytes. Angew. Chem. Int. Ed..

[95] Balabushevich N.G., Lopez de Guerenu A.V., Feoktistova N.A., Skirtach A.G., Volodkin D. (2016). Protein-containing multilayer capsules by templating on mesoporous CaCO_3_ particles: POST- and PRE-loading approaches. Macromol. Biosci..

[96] Hou X., Guo W., Jiang L. (2011). Biomimetic smart nanopores and nanochannels. Chem. Soc. Rev..

[97] Sun Z., Liao T., Zhang Y., Shu J., Zhang H., Zhang G. (2016). Biomimetic nanochannels based biosensor for ultrasensitive and label-free detection of nucleic acids. Biosens. Bioelectron..

[98] Fang R., Zhang H., Yang L., Wang H., Tian Y., Zhang X., Jiang L. (2016). Supramolecular self-assembly induced adjustable multiple gating states of nanofluidic diodes. J. Am. Chem. Soc..

